# Diversity of Leaf Glands and Their Putative Functions in Rhamnaceae Species

**DOI:** 10.3390/plants12213732

**Published:** 2023-10-31

**Authors:** Lucas Iwamoto, Thales Augusto Vicentini, Felipe Paulino Ramos, Carimi Cortez Ribeiro, Simone Pádua Teixeira

**Affiliations:** 1 Departamento de Ciências Farmacêuticas, Faculdade de Ciências Farmacêuticas de Ribeirão Preto, Universidade de São Paulo, Ribeirão Preto 14040-903, Brazil; lucas.iwamoto@usp.br (L.I.); vicentinithales@usp.br (T.A.V.); felipepramos@usp.br (F.P.R.); carimicortez@hotmail.com (C.C.R.); 2 Departamento de Biologia, Faculdade de Filosofia, Ciências e Letras de Ribeirão Preto, Universidade de São Paulo, Ribeirão Preto 14040-901, Brazil

**Keywords:** *Colubrina glandulosa*, extrafloral nectary, *Gouania polygama*, plant–animal interaction, *Rhamnidium elaeocarpum*, secretory cavity, secretory duct

## Abstract

Leaf glands are found in many Rhamnaceae species, the buckthorn family, and are frequently used in taxonomic studies of the group, especially because they are easily visible to the naked eye. Despite the many records and extensive use in the taxonomy of the family, few studies deal with the classification of these glands and their roles for the plant. Thus, this study aimed to unravel the type, functioning, and putative functions of the leaf glands of three Brazilian forest species: *Colubrina glandulosa* Perkins, *Gouania polygama* (Jacq.) Urb., and *Rhamnidium elaeocarpum* Reissek. Leaves were collected and processed for surface, anatomical, histochemical, and ultrastructural analyses. In addition, the presence of visitor animals was registered in the field. The leaf glands of *C. glandulosa* and *G. polygama* are defined as extrafloral structured nectaries due to their anatomical structure, interaction with ants, and the presence of reduced sugars and of a set of organelles in the secretory cells. The unusual mechanism of nectar release and exposure in an apical pore stands out in *G. polygama*. The glands of *R. elaeocarpum* are ducts or cavities that secrete phenolic oil resin. Their presence is an atypical condition in the family, although they are often confused with mucilage reservoirs, much more common in Rhamnaceae. The extrafloral nectary, secretory cavity, and duct are associated with plant protection against phytophages, either by attracting patrol ants or by making the organs deterrent. Our data, combined with other previously obtained data, attest to the great diversity of gland types found in Rhamnaceae species.

## 1. Introduction

Secretory structures or glands are characterized as cells or groups of cells that produce, store, and/or release specific substances by cell disruption or by cellular transport [1]. They can be classified into two classes according to the composition of the exudate: (i) those that secrete little modified or unmodified substances, such as hydathodes, salt glands, and nectaries, supplied directly or indirectly by vascular bundles, and (ii) those that secrete substances modified by the secretory tissue, such as glands that produce hydrophilic or lipophilic substances [1].

Many plants have some type of secretory structure, showing great diversity even in older divergent groups, such as ferns, which have mucilage- or tannin-secreting cavities and ducts [2]. There are even reports of leaf nectaries in *Pteridium* species that allow the plant to interact with arthropods for protection against herbivores [3], and minute secretory trichomes in rhizomes and leaves of *Polypodium* species [4]. Glands are also present in gymnosperms, which often have resin ducts in some genera (*Pinus*, *Picea*, *Larix*, *Pseudotsuga*), and which can even be formed as a strategy of defense after injury in others (*Abies*, *Cedrus*, *Tsuga*, *Pseudolarix*) [5,6]. These glands often provide key characters for the taxonomic delimitation of species and genera [7].

In angiosperms, secretory structures are also present and have been extensively reported in the literature, especially those related to the interaction of animals with flowers for pollination. One of the main glands involved in this interaction is the nectary, a sugary solution-secreting structure that acts as a nutritional resource for pollinators, thus encouraging interaction [8]. The nectaries have been classified in different ways over the years, initially based on the location criterion, i.e., as floral when present in the floral organs, and as extrafloral when present in other organs [9]. Extrafloral nectaries act as a defensive measure against herbivory by providing nectar to ants, wasps, or spiders, which in exchange will protect against other herbivores, but they can also have the function of distracting ants from other attractors, such as the flower. They can even serve as a preventive measure against the association of hemipterans and ants [10]. Another classification considers the function of such structures, with a nectary called reproductive when it is directly related to pollination, in general present in flowers, inflorescences and fruits; and extra-reproductive when it is not directly related to pollination and is strictly located in vegetative organs [11]. The nectary can also be classified as structured or non-structured according to its internal morphology [12].

Like extrafloral nectaries (EFNs), glands known as feeding bodies (“food bodies”) have the function of interacting with animals, with the plant providing nutrients and animals providing protection against other herbivores, being characterized by the accumulation of proteins, lipids, and carbohydrates [13]. Although these substances are abundant in other plant structures, they are much more concentrated in the food bodies, occupying almost the entire intracellular environment [14]. The morphology of these structures includes the presence of unicellular or multicellular trichomes or is characterized by protuberances mainly consisting of parenchyma [15]. Resin ducts have a similar defensive function by producing and storing oleoresin, which is toxic to herbivores and pathogens but is only released when the plant is wounded [16]. In *Pinus contorta* Douglas ex Loudon (Pinaceae), for instance, the size of such structures was the main survival factor during mountain pine beetle outbreaks [17].

Rhamnaceae, also known as the buckthorn family, is one of the nine families in the Rosales order, and comprises about 66 genera and 1100 species such as herbs, trees, and vines of worldwide distribution, but mainly occurring in tropical and subtropical regions [18,19]. Some of these species are capable of nitrogen fixation [20] and others are of economic importance, producing edible fruits and wood, and being used as ornamental plants [21,22], besides having medicinal uses, as recorded in ancient texts [23,24].

Most Rhamnaceae species exhibit some type of secretory structure both in the flower and leaf [13,25,26,27,28,29,30,31], like nectaries, phenolic and mucilage idioblasts, oil resin cavities and ducts, mucilage cavities and ducts, and colleters and glandular trichomes. Although a large number of species appear in the excellent compendiums by Gemoll (1902) [25] and Solereder (1908) [26], the glands are not characterized in detail and, therefore, the definition of types is dubious. This lack of in-depth information invalidates the establishment of phylogenetic relationships between the groups, making the taxonomic circumscription of taxa difficult, since the occurrence and position of glands in the leaves have been widely used with taxonomic value. A few studies have unraveled the types of glands, such as those by Buono et al. (2008) [13] that characterizes the leaf glands of *Hovenia dulcis* as food bodies instead of extrafloral nectaries, as previously classified; and by Wollenweber et al. (2004) [31] that defined the glandular leaf teeth of *Ceanothus* species as glandular trichomes.

*Colubrina*, *Gouania*, and *Rhamnidium* are examples of genera in which the occurrence and location of leaf glands are characters used in species descriptions and in taxonomic identification keys. *Colubrina* species have conspicuous leaf glands at the base and/or close to the margin of the leaf blade; *Gouania* species have conspicuous and pubescent glands on the leaf teeth; and *Rhamnidium* species have punctiform and scattered glands throughout the leaf blade [32,33,34]. However, in none of these genera were the leaf glands structurally or chemically defined.

Thus, considering the divergences or lack of in-depth information regarding the glands found in the leaf of Rhamnaceae species, and their ecological and medicinal importance, the objective of the present study was to unveil the type and functioning of the leaf glands in Brazilian forest species of three different genera, selected due to previous records of the presence of leaf glands: *Colubrina glandulosa* Perkins, *Gouania polygama* (Jacq.) Urb. (=*Gouania virgata* Reissek), and *Rhamnidium elaeocarpum* Reissek. Our proposition is that the visible glands on the leaves of the three species under evaluation differ from one another, thus confirming the considerable diversity of secretory structures previously identified within the Rhamnaceae family. Consequently, we anticipate the presence of extrafloral nectaries, colleters, hydathodes, and/or food bodies in these leaves. Putative functions of these glands in plant–arthropod interaction are raised and discussed.

## 2. Results

### 2.1. Colubrina glandulosa

**Figure 1:**
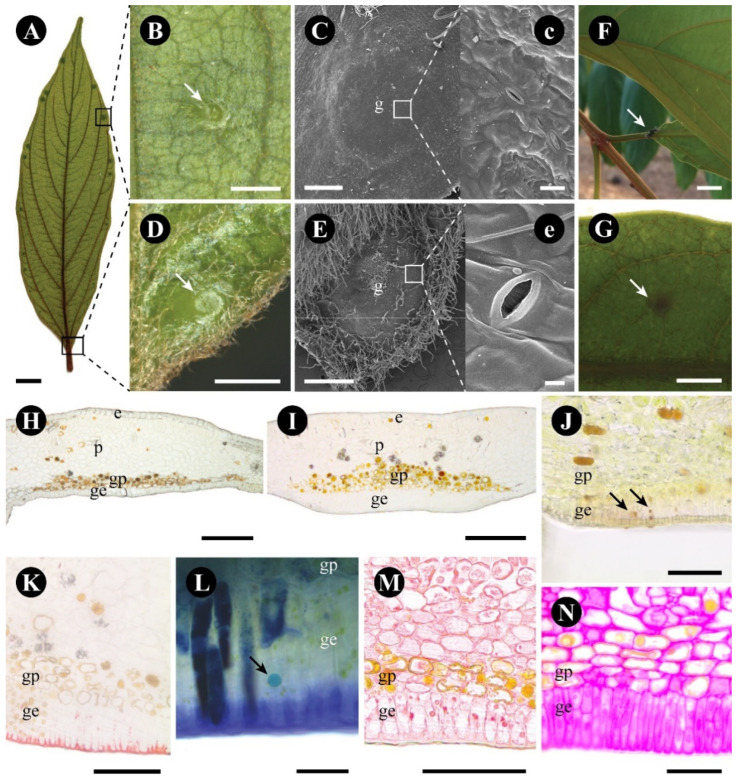
Morphology of a *Colubrina glandulosa* leaf gland. (**A**) Glands (squares) distributed over the abaxial side of the leaf. (**B**) Detail of the gland on the abaxial face, in the region of the leaf margin. (**C**) Surface of the protuberant margin gland, and its stomata in detail (**c**). (**D**) Detail of the gland on the abaxial surface, in the region of the leaf base. Note the tector trichomes around the gland. (**E**) Surface of the protuberant base gland, and its stomata in detail (**e**). (**F**) Field observation, with an ant interacting with the gland area (arrow). (**G**) Detection of reducing sugar (arrow), with Fehling reagent. (**H**,**I**) Anatomical sections (not stained) of the gland in the base (**H**) and in the margin (**I**) of the leaf, showing the glandular epidermis (ge) and the glandular parenchyma (gp). (**J**–**N**). Anatomical sections (stained) of the glands, showing positive reactions in the glandular epidermal cells for reducing sugar (arrow) with Fehling reagent (**J**), for lipids with Sudan IV (**K**), for terpenes (arrow) with NADI reagent (**L**), for proteins with Xylidine ponceau (**M**), and for neutral polysaccharides with PAS reagent (**N**). Scale bars: (**A**,**F**) = 1 cm; (**B**,**D**,**E**,**G**) = 1 mm; (**C**,**H**,**I**) = 200 µm; (**J**,**M**,**K**) = 100 µm; (**N**) = 50 µm; (**c**,**L**) = 20 µm. Abbreviations: e = non-glandular epidermis, g = gland, ge = glandular epidermis, gp = glandular parenchyma, p = non-glandular parenchyma.

The leaf glands of *C. glandulosa* are easily identified by their dark coloration (Figure 1A) and discoid shape. They occur in the leaf margin and base (Figure 1A), and are conspicuous on the abaxial surface (Figure 1B–E) and inconspicuous in the adaxial surface. Through the development of the leaf, the appearance of the glands is asynchronous beginning with the pair of glands in the leaf base and then the glands in the margin, without a specific sequence; when fully developed, each leaf presents around 25 glands in total. The glands in the leaf base and margin are structurally similar, except for the presence of tector trichomes around the margin gland (compare Figure 1B,C with Figure 1D,E).

There is a large concentration of modified stomata on the gland area (Figure 1c,e). A range of animals visited the leaf throughout the day, but usually just passing through; only ants were consistently present and visited the gland area during our observations (Figure 1F). A small amount of transparent and viscous exudate, positively reacting for reducing sugar with Fehling reagent (Figure 1G), was observed.

**Figure 2:**
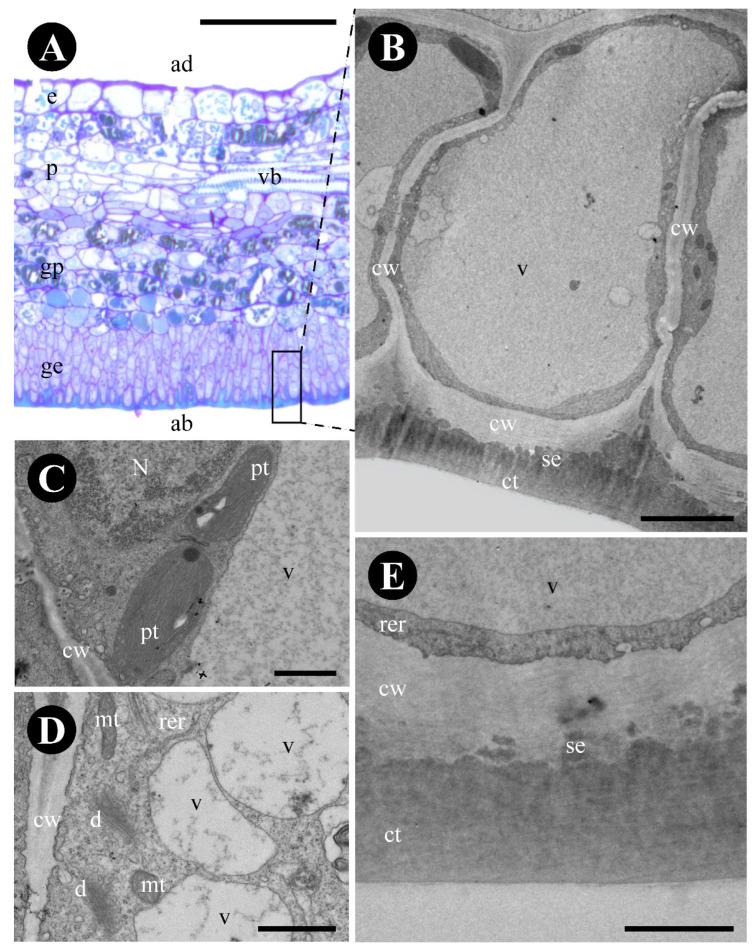
Anatomy and ultrastructure of the epidermal cells of *Colubrina glandulosa* foliar nectary. (**A**) Anatomical section stained with toluidine blue, showing the glandular epidermis (framed). (**B**–**E**) Ultrastructure of the glandular epidermal cells. (**B**) Epidermal glandular cell showing a voluminous vacuole, thin walls, cuticle, and secretion in the subcuticular space. (**C**) Detail of plastids with amilliferous and lipidic inclusions. (**D**). Detail of mitochondria, dictyosomes, and rough endoplasmic reticulum. (**E**) Detail of periclinal cell wall and cuticle. Note that the secretion intersperses the cell wall and the cuticle. Scale bars: (**A**) = 100 µm; (**B**) = 5 µm; (**C**–**E**) = 1 µm. Abbreviations: ab = abaxial side, ad = adaxial side, ct = cuticle, cw = cell wall, d = dictyosome, e = epidermis, ge = glandular epidermis, gp = glandular parenchyma, mt = mitochondria, N = nucleus, p = parenchyma, pt = plastid, rer = rough endoplasmic reticulum, se = secretion, v = vacuole, vb = vascular bundle.

Both margin and base leaf glands consist of a uniseriate palisade glandular epidermis and subepidermal glandular parenchyma (Figure 1H–N and Figure 2A), naturally stained brown (Figure 1H,I) and supplied by xylem and phloem elements (Figure 2A).

The glandular epidermis is cuticularized and has thin-walled cells (Figure 2B). Terpenes (Figure 1L), protein bodies (Figure 1M), and reducing sugars (Figure 1G,J,N) were detected in the cytoplasm (Table 1). The produced substances were observed inside the large central vacuole, between the plasma membrane and the cell wall and in subcuticular space (Figure 2B–D). The cytoplasm of the glandular epidermal cells has plastids with plastoglobuli, mitochondria, rough endoplasmic reticulum, vesicles, and ribosomes (Figure 2C–E).

**Figure 3:**
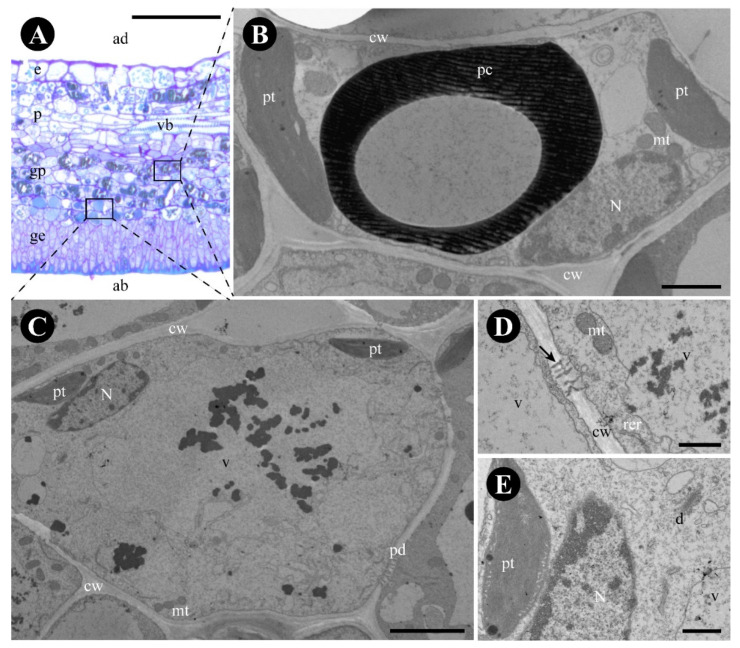
Anatomy and ultrastructure of the glandular parenchyma of *Colubrina glandulosa* foliar nectary. (**A**) Anatomical section stained with toluidine blue, showing the nectariferous parenchyma (framed). (**B**–**E**) Ultrastructure of the glandular parenchyma cells. (**B**) Phenolic cell of the delimiting layer. (**C**) Sugar-secreting cell. Note the large vacuole with granular material and plastids with plastoglobuli. (**D**) Detail of the plasmodesmata (arrow). (**E**). Detail of dictyosome and of a plastid with plastoglobuli. Scale bars: (**A**) = 100 µm; (**B**) = 5 µm; (**C**) = 2 µm; (**D**,**E**) = 1 µm. Abbreviations: ab = abaxial side, ad = adaxial side, cw = cell wall, d = dictyosome, e = epidermis, ge = glandular epidermis, gp = glandular parenchyma, mt = mitochondria, N = nucleus, p = parenchyma, pd = plasmodesmata, pt = plastid, rer = rough endoplasmic reticulum, v = vacuole, vb = vascular bundle.

The glandular parenchyma cells are isodiametric and have thin uniform walls (Figure 3A). Phenolic compounds were found in the cytoplasm of the glandular parenchyma (Figure 1H–I and Figure 3B), forming an endodermic layer delimiting the gland area and the rest of the leaf (Figure 1H–I), in addition to having crystals in the glandular and non-glandular parenchyma without a specific arrangement. Non-phenolic cells of the glandular parenchyma contain a large vacuole, with abundant granular material (Figure 3C), a peripheric nucleus (Figure 3C), plastids with lipidic inclusions, mitochondria, rough endoplasmic reticulum, active dictyosomes, and plasmodesmata (Figure 3C–E).

### 2.2. Gouania polygama

**Figure 4:**
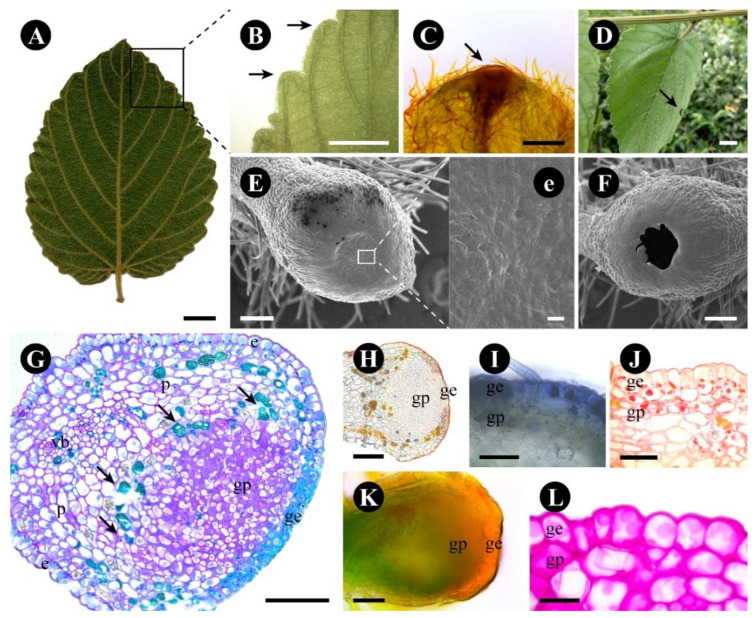
Morphology of *Gouania polygama* foliar nectary. (**A**,**B**) Entire leaf, abaxial side, showing glands (arrow) distributed along the leaf margin teeth. (**C**) Detail of a gland in a leaf margin tooth stained with Fehling reagent for reducing sugar detection. (**D**) Ant in a glandular region (arrow). (**E**,**F**) Surface of glands before (**E**) and after (**F**) secretion release. Note that a pore is formed in (**F**). (**e**) Detail of the smooth glandular surface. (**G**–**L**) Anatomical longitudinal sections showing an overview of the gland ((**G**)—stained with toluidine blue), ((**H**)—stained with Sudan IV), terpenic droplets ((**I**)—stained with Nadi reagent), protein granules ((**J**)—stained with Xylidine ponceau), reducing s–gars ((**K**)—stained with Fehling reagent), and starch grains ((**L**)—stained with PAS reagent). Scale bars: (**A**,**D**) = 1 cm; (**B**) = 5 mm; (**C**) = 500 µm; (**E**–**K**) = 100 µm; (**I**,**J**) = 50 µm; (**L**) = 20 µm; (**e**) = 10 µm. Abbreviations: e = non-glandular epidermis, ge = glandular epidermis, gp = glandular parenchyma, p = non-glandular parenchyma, vb = vascular bundle.

The leaf glands of *G. polygama* are easily identified by their lighter coloration (Figure 4A). They are disc-shaped, protuberant, and occur on all leaf margin teeth, coinciding with the end of the secondary veins (Figure 4B,C), each leaf presenting around 30 glands, that are already present in the leaf primordia and increase in size as the leaf advances in its development.

A transparent and viscous exudate, released in small amounts, was detected during observations carried out on the plants. Several animals were observed around and on the plant throughout the day, such as coleoptera and arachnids, with no preference for a specific region. Patrolling ants were observed on the surface of the plant and towards the leaves, mainly in the region of the apex of the leaf ridges where the glands are located (Figure 4D).

The gland surface is smooth with no stomata (Figure 4E,e), the outer region of the gland is elevated, forming a pit at its tip (Figure 4E), and, at the time of the releasing process, the central region ruptures, forming a large pore but leaving the adjacent tissues intact (Figure 4F). The adjacent epidermis is formed by higher cells (Figure 4G); stomata are scarce.

The gland has a uniseriate glandular epidermis, a subepidermal glandular parenchyma formed by more than five cell layers supplied by xylem and phloem elements, and a layer of phenolic and crystalliferous cells forming a barrier delimiting the gland area from the rest of the leaf (Figure 4G).

**Figure 5:**
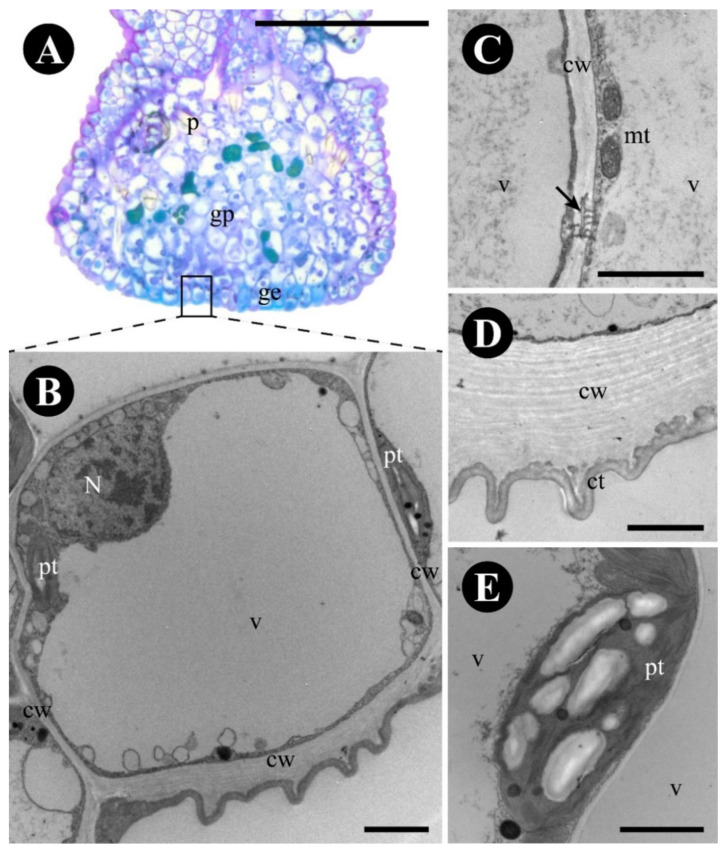
Anatomy and ultrastructure of the epidermal cells of *Gouania polygama* foliar nectary. (**A**) Anatomical longitudinal section stained with toluidine blue, showing the glandular epidermis with lignified walls (framed) and the glandular parenchyma. (**B**–**E**) Ultrastructure of the glandular epidermal cells. (**B**) Overview of a cell showing the walls, the peripheral nucleus, and the large central vacuole. (**C**) Detail of mitochondria and plasmodesmata (arrow). (**D**) Detail of the outer periclinal cell wall and ornate cuticle. (**E**) Detail of a plastid with several starch grains and plastoglobuli. Scale bars: (**A)** = 200 µm, (**B**–**E**) = 2 µm. Abbreviations: ct = cuticle, cw = cell wall, ge = glandular epidermis, gp = glandular parenchyma, mt = mitochondria, N = nucleus, p = parenchyma, pt = plastid, v = vacuole.

The glandular epidermis has two types of cells: central quadrangular cells with thick lignified walls (Figure 4G), especially the outer periclinal ones, and surrounding elongated cells with pectocellulosic walls (Figure 4G,H), cuticle (Figure 4H), and stomata (Figure 4e). Terpenes (Figure 4I), protein bodies (Figure 4J), and reducing sugars (Figure 4C,K,L) were detected in the cytoplasm of the glandular epidermal cells, especially in the surrounding cells (Table 1). The central region of the gland has a thin ornate cuticle (Figure 5D–E), lignified cells with a central large vacuole, a peripheric nucleus, mitochondria, and plastids (Figure 5A–E).

**Figure 6:**
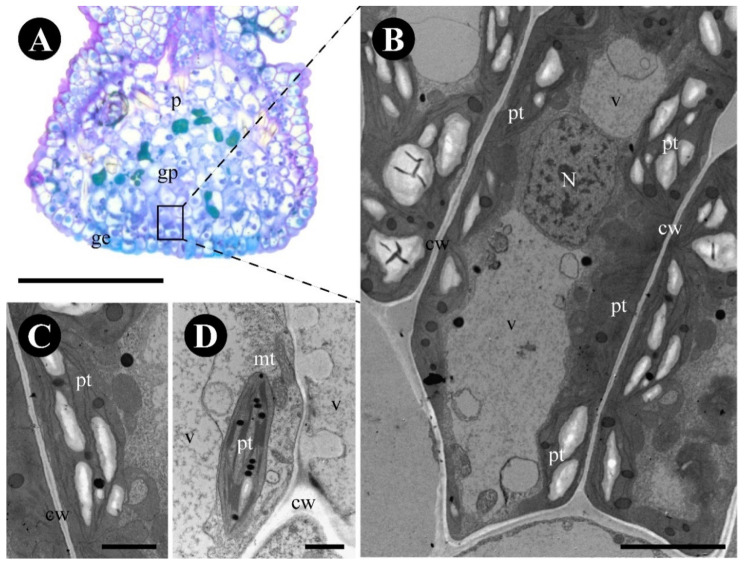
Anatomy and ultrastructure of the glandular parenchyma cells of *Gouania polygama* foliar nectary. (**A**) Anatomical longitudinal section stained with toluidine blue, showing the glandular epidermis with lignified walls and the glandular parenchyma (framed). (**B**–**D**) Ultrastructure of the glandular parenchyma cells. (**B**) Overview of a sugar-secreting cell showing the central nucleus, large vacuoles, and several amyloplasts with plastoglobuli. (**C**) Detail of an amyloplast with plastoglobuli. (**D**) Detail of mitochondria and a plastid with plastoglobuli. Scale bars: (**A**) = 200 µm, (**B**) = 5 µm, (**C**,**D**) = 2 µm. Abbreviations: cw = cell wall, ge = glandular epidermis, gp = glandular parenchyma, mt = mitochondria, N = nucleus, p = parenchyma, pt = plastid, v = vacuole.

The subepidermal glandular parenchyma cells are isodiametric to elongated (Figure 6A,B), and have thin walls of uniform thickness, central nuclei, large vacuoles with granular material, rough endoplasmic reticulum (Figure 6A–D), and numerous plastids with starch grains and plastoglobuli (Figure 6C,D).

**Figure 7:**
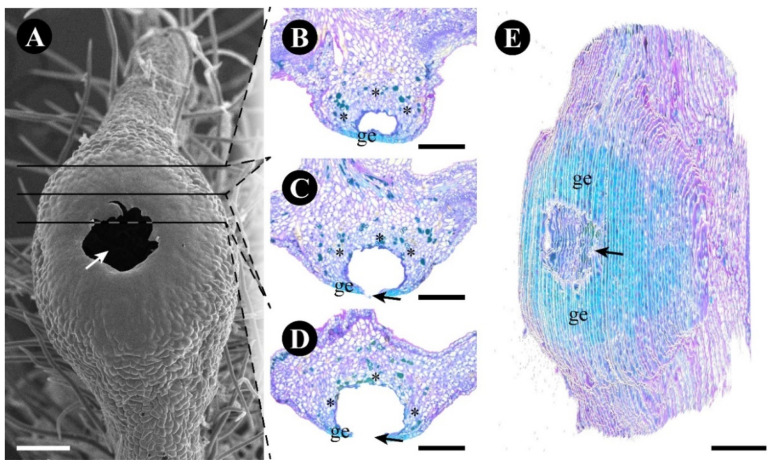
Three-dimensional reconstruction of a *Gouania polygama* leaf gland. (**A**) Surface of the gland after nectar release, showing the open gland (arrow). (**B**–**D**) Anatomical sections showing an overview of an open gland (arrow), stained with toluidine blue. (**E**) Three-dimensional reconstruction of the gland with serial sections of the open gland (arrow) showing the external structure, stained with toluidine blue. Scale bars: (**B**–**E**) = 200 µm; (**A**) = 100 µm. Abbreviations: ge = glandular epidermis; * = crystalliferous and phenolic cells layer.

The large pore of the gland is formed by the rupture of the epidermal central area formed of lignified epidermal cells (Figure 4 and Figure 7). This rupture causes the glandular parenchyma exposure that disintegrates, leaving only the delimiting phenolic and crystalliferous cells intact, thus forming a large internal space (Figure 7). This process does not occur simultaneously in all glands in the same leaf.

### 2.3. Rhamnidium elaeocarpum

**Figure 8:**
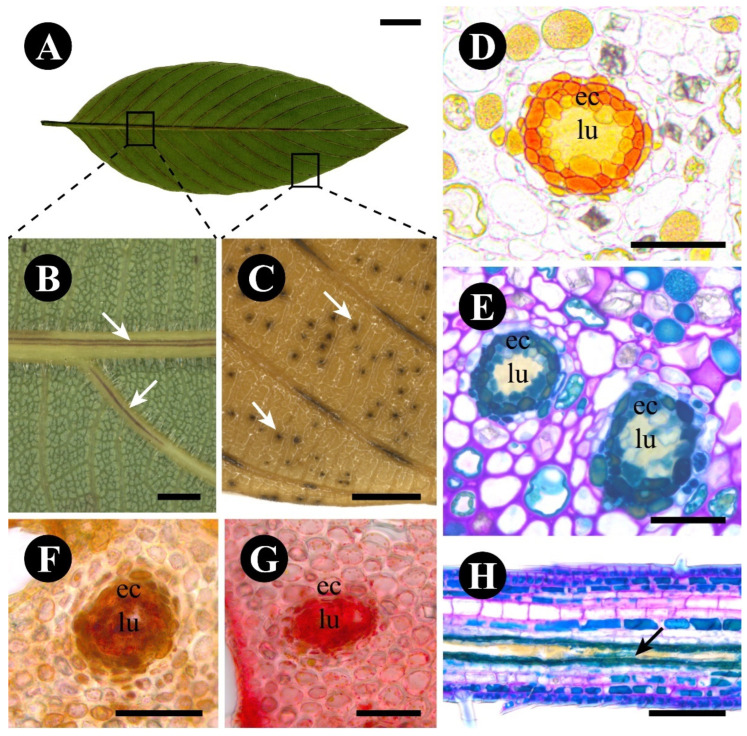
Morphology of *Rhamnidium elaeocarpum* leaf glands: secretory duct and cavity. (**A**) Entire leaf, abaxial view, showing the regions where the glands are found. (**B**,**C**) Dark glands (arrows—duct) found in the veins (**B**, fresh sample) and in the veins (duct) and interveinal (arrows—cavity) regions (**C**, fixed sample). (**D**–**H**) Anatomical sections of the leaf glands showing the natural color of the secretion (**D**), and positive reactions for phenolic compounds with toluidine blue (**E**), for lipids with Sudan IV (**F**), and for proteins with Xylidine ponceau (**G**). (**H**) Longitudinal section showing the elongated lumen of a secretory duct (arrow). Scale bars: (**A**) = 1 cm; (**B**) = 1 mm; (**C**) = 2 mm; (**D**–**H**) = 100 µm. Abbreviation: ec = epithelial cell, lu = lumen.

The leaf glands of *R. elaeocarpum* are easily identified by their dark coloration (Figure 8A,B), especially after the samples are fixed (Figure 8C). They are elongated in the midrib and veins (Figure 8B,C), and dotted in the interveinal regions (Figure 8C), without displaying a particular pattern. A range of animals visited the leaf throughout the day, usually passing through and not interacting with the leaf gland, but it was apparent that the leaves had a lower herbivory rate, independent of the weather or time of day, with most collected leaves showing no signs of injury.

The glands are internal and do not protrude outside of the leaf surfaces. They have a ring of secretory epithelial cells delimiting an internal lumen, the content of which is naturally yellow (Figure 8D). The epithelial cells are naturally orange and surrounded by a parenchymatic sheath (Figure 8D). Phenolic compounds were detected in the cytoplasm of the epithelial cells (Figure 8E), in addition to lipids (Figure 8F) and protein bodies (Figure 8G) inside the lumen and in the cytoplasm of epithelial cells. In the interveinal regions, the lumen is spherical (Figure 8C), and the gland is named secretory cavity. In the midrib and veins, the lumen is elongated (Figure 8H); thus, the gland resembles a secretory duct.

**Figure 9:**
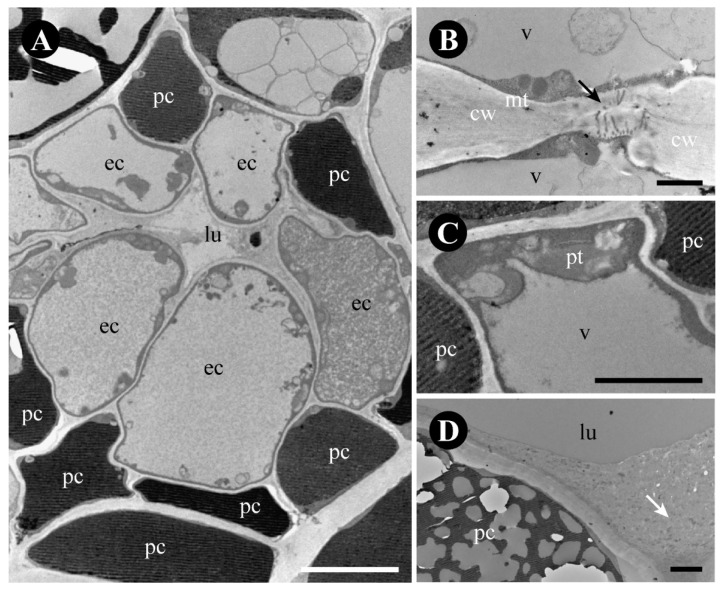
Ultrastructure of *Rhamnidium elaeocarpum* secretory cavity cells. (**A**) Overview of a secretory cavity showing the epithelial cells and the sheath phenolic cells. Note the secretion within the lumen. (**B**) Detail of the plasmodesmata (arrow) between two epithelial cells. (**C**) Detail of a plastid. (**D**) Detail of a phenolic sheath cell and of the lumen, with secretion (arrow). Scale bars: (**A**) = 5 cm; (**B**) = 2 mm; (**C**,**D**) = 1 µm. Abbreviation: cw = cell wall, ec = epithelial cell, lu = lumen, mt = mitochondria, pc = phenolic compound, pt = plastid, v = vacuole.

The epithelial cells are isodiametric with thin walls (Figure 9A) and plasmodesmata (Figure 9B). They include a central nucleus, a large vacuole filled with granular content (Figure 9A), small mitochondria (Figure 9B), and plastids at the periphery of the cell (Figure 9C). The cells of the parenchymatic sheath have a large vacuole filled with phenolic compounds (Figure 9A). The lumen has a build-up of secretion similar to that stored into the vacuole of the epithelial cells (Figure 8D,F,G and Figure 9D).

## 3. Discussion

### 3.1. The Foliar Glands of Colubrina glandulosa and Gouania polygama Are Extrafloral Nectaries

The leaf glands of *C. glandulosa* and *G. polygama* are extrafloral nectaries (EFNs), based on the following data: (i) the presence of reducing sugars detected by positive reactions with Fehling’s reagent in the exudate of the gland; (ii) the anatomical structure of the glands, with a secretory epidermis with modified stomata and a vascularized subepidermal secretory parenchyma delimited by an endodermoid phenolic layer, in addition to crystals in the case of *G. polygama*; (iii) parenchyma cells with amyloplasts, in the case of *G. polygama*, and rich in ribosomes and mitochondria, in the case of *C. glandulosa*; (iv) the presence of ants in the regions where the glands are located in the leaves [8]. These EFNs can be further classified as structured, extra reproductive, and vascularized, according to the criteria mentioned by Fahn (1979) [1].

Comparisons between *Colubrina glandulosa* and *Gouania polygama* regarding ecological relationships should be analyzed with greater attention, since in the present study the number of hours of observation was not sufficient for in-depth conclusions, in addition to the fact that the plants were located in very different environments, managed gardens for *C. glandulosa* and a semi-deciduous seasonal native forest area for *G. polygama*, which may have influenced their interactions. The asynchronous development of EFNs along the leaf lamina in *C. glandulosa* and *G. polygama*, i.e., starting at different times in the same leaf in *C. glandulosa* and starting together and finishing at different times in the same leaf in *G. polygama*, is probably related to the permanent protection of the plant against phytophages. Continuously providing nectar glands can attract ants through the development of the organ, thus increasing the plant survivability [35].

The presence of terpenes in the glandular cells of *C. glandulosa* and *G. polygama* extrafloral nectaries (see Table 1) is surprising and should be highlighted, since it is a condition rarely reported in nectaries in general [8]. Several functions can be proposed for the presence of terpenes in nectary cells, such as protection against herbivory in the nectary areas, since the loss of such a structure can be harmful to the plant, as can be seen regarding the oil gland of *Bothriochloa alta* [36] or the EFNs of *Cydista* species [37]. Volatile oils may also attract animals, mainly ants, providing olfactory attraction in addition to visual attraction [38]. Another proposition is that terpenes may not be directly linked to EFN function but may act as thermotolerant agents that help plant photosynthesis [39]. However, considering that the terpene droplets found in both species were in the EFN-secreting epidermis, this last proposition is unlikely.

Most studied species of Rhamnaceae have floral nectaries [30,40]. *Colubrina glandulosa*, in particular, exhibits a very evident, discoid, yellow floral nectary [30], thus being anatomically similar to the EFNs described in the present study, displaying a rough surface covered by a uniseriate palisade epidermis with stomata, a vascularized glandular subepidermal parenchyma, and a phenolic endodermoid layer. The floral and extrafloral nectaries of *Gouania polygama* are also similar, mainly with respect to the subepidermal parenchyma, which has phenolic and crystalliferous cells, but they differ regarding the presence of stomata in the glandular epidermis, since the EFNs of this species have a smooth surface without stomata. One way to delimit and protect the nectariferous area, in addition to preventing the reabsorption of substances that will be transported by the vascular system, is the formation of an endodermoid sheath, commonly through the accumulation of phenolic compounds, mucilage, and/or crystals, as described for *Polygonum* species [41] and *Hibiscus rosa-sinensis* [42]. Both EFNs have an endodermoid sheath formed by a layer of phenolic cells, and in the case of *G. polygama*, there is also the presence of crystalliferous cells, which make it more easily distinguishable.

An Interesting difference between the EFNs of *C. glandulosa* and *G. polygama* concerns nectar release. In *C. glandulosa*, the nectary is typical, with a thick cuticle and modified stomata, through which the nectar is directly released into the external environment [8]. This release could also occur via the cuticle, as in *Maxillaria coccinea* (Orchidaceae) [43], in which the nectar is found in the subcuticular space of the epidermal cells. On the other hand, the nectary of *G*. *polygama* has an unusual structure and function, which is reflected on nectar release.

In an attempt to understand the functioning of the interesting *G*. *polygama* EFN, we elaborated the following propositions. (a) The nectar may be released directly through the glandular epidermis, which, despite being thick-walled, exhibits accumulation of nectar in the subcuticular space. The lignification of these walls seems to make this form of release unfeasible, but, on the other hand, the cuticle is thin in this region. (b) Nectar exposure may be similar to pollen presentation in the poricidal anther opening mechanism reported, for example, in *Miconia* species (Melastomataceae) [44]. The presence of lignified cells may form a region of weakness, causing the disruption of the glandular epidermal surface and the formation of a large pore at the apex of the gland, which is delimited inside by the endodermal phenolic/crystalliferous layer that isolates the internal structure of the leaf from the external ambient created by the pore formation. The nectar may be released via stomata present in the adjacent epidermis and/or via degradation of the glandular parenchyma cells (holocrine secretion), and may accumulate inside the pore. In this way, the nectar would be exposed to visiting animals such as ants.

### 3.2. The Foliar Glands of Gouania polygama and Those of Rosaceae Species

The location of leaf glands at the apex of leaf teeth in G. polygama coincides with that of *Prunus species* [45], *Mespilus germanica* [46], and *Rosa lucieae* [47]. According to the authors, the presence of sticky exudate and the loss of the secretory head of the glands after exudate release in these species indicate that they are colleters. However, our data obtained from the *G. polygama* gland reveal morphological and histochemical characteristics different from those found in Rosaceae species.

First, the anatomy does not conform to the typical description of a colleter, according to Fahn (1979) [1]. Colleters are prominent secretory structures formed from protodermis and ground meristem cells, typically found in developing plant organs. The classic type of colleter consists of palisade epidermal cells delimiting a parenchyma stalk. Its exudate mainly contains acidic polysaccharides and lipids. Second, the positive reaction for reducing sugars (Fehling’s reagent), the presence of parenchyma cells filled with amyloplasts, similar to those found in nectariferous parenchyma, and the presence of ants in the region of the glands provide evidence for the presence of extrafloral nectaries rather than colleters. Finally, our observations show that the lysis of the cells forming the pore occurs in a way that exposes the exudate in an internal space in the leaf to the animal, similar to a cup, which is also observed in floral nectaries of Rhamnaceae species (Ribeiro et al., 2022 [30]). It is interesting to note that both colleters and extrafloral nectaries occur in the leaves of *Prunus* species [45], indicating that the presence of colleters does not exclude the presence of extrafloral nectaries in this organ.

Comparing the foliar glands of Rhamnaceae species to those of Rosaceae is crucial for making taxonomic decisions and for the evolutionary study of such glands, especially considering their phylogenetic proximity. These families are both included in the Rosales order [48]. Therefore, future studies aiming at identifying colleters and detailing their cellular structure in Rhamnaceae can provide valuable insights into the questions raised.

### 3.3. The Foliar Glands of Rhamnidium elaeocarpum Are Secretory Ducts and Cavities

The leaf glands of *Rhamnidium elaeocarpum* are internal structures named secretory cavities or ducts [1], depending on their location. The secretory ducts occur in the region of the veins and are characterized by an elongated lumen, where the exudate is released, delimited by a secretory epithelium. The secretory cavities occur in the interveinal region, and the epithelium delimits a spherical, isodiametric lumen. Ducts and cavities have also been detected in the floral organs, in the pedicel (duct), in the gynoecium (cavity), and in the hypanthium [29]. Such floral and foliar glands have a similar content with a vibrant green color when stained with toluidine blue (pH 5.8), indicating the presence of resin and phenolic compounds, in addition to similar results for the detection of lipids. These results suggest that these glands may be present throughout the plant’s body, and may also perform similar functions such as defense against phytophagous insects, as shown by the low rate of herbivory of *R. elaeocarpum* plants in field observations (personal observation). Other structures for defense against herbivory [49] recorded in the leaves of this species are volatile oil-secreting idioblasts and large crystalliferous cells.

Cavities and/or ducts mainly secreting mucilage have been recorded in about 100 species of Rhamnaceae (see Table S1 by Ribeiro et al., 2021 [29]). However, these structures are not in fact ducts or cavities, considering Fahn’s concept (1979), but mucilage reservoirs, formed by the lysis of the walls of several grouped mucilage cells, a condition commonly described in species of Rhamnaceae [50,51,52,53], including *R. elaeocarpum* [29]. Within the Rhamnoid clade, to which *Rhamnidium* belongs, among the many species with records of secretory structures, leaf ducts or cavities secreting mucilage were reported only for *Rhamnus diffusa* and *Rhamnidium glabrum* [54]. We believe that such ducts/cavities described by Herzog (1903) for *R. diffusa* are actually mucilage reservoirs, as in the species mentioned above, but that *R. glabrum* deserves further investigation, in order to verify if there are cavities and ducts like its closely related species *Rhamnidium elaeocarpum* (present study).

No secretory ducts or cavities were found in the sister group of Rhamnaceae, which is composed of three families, namely, Barbeyaceae, Dirachmaceae, and Elaeagnaceae, and also in species of Cannabaceae, Moraceae, Urticaceae, and Ulmaceae, that compose the Urticoid clade. All these families belong to the order Rosales [48]. It is, therefore, a fact that the distinction between secretory ducts/cavities and mucilage reservoirs is poorly understood in the literature, which allows us to consider that the presence of secretory ducts and cavities is an uncommon condition in Rhamnaceae.

## 4. Materials and Methods

Representatives of the study species are arboreal (*C. glandulosa* and *R. elaeocarpum*) or woody liana (*G. polygama*), and occur mainly in phytogeographical domains of the Amazon and Atlantic Forests, and the Cerrado [55].

Collections of the former two species were carried out from adult specimens located in the Sao Paulo University campus, Ribeirão Preto, Brazil (21°10′19.5″ S 47°50′47.7″ W and 21°10′09.9″ S 47°51′03.1″ W, respectively), whereas those of *G. polygama* were carried out from adult specimens located around Mata de Santa Tereza, Ribeirão Preto, Brazil (21°12′46.6″ S 47°50′42.9″ W). The vouchers of the three species were identified and deposited in the SPFR herbarium under the following voucher numbers: *C. glandulosa*—SPFR 17191, *G. polygama*—SPFR 17188, and *R. elaeocarpum*—SPFR 17158.

Samples of young and fully expanded leaves were collected in the morning between 7:00 and 10:00 a.m. Part of the samples was observed and photographed under a Leica MDG 34 (Wetzlar, Germany) stereomicroscope with a Leica DFC 425 digital camera. The other part was fixed in neutral buffered formalin solution for 24 h at room temperature, washed in phosphate buffer solution, pH 6.8, gradually dehydrated in an alcohol series, and stored in 70% alcohol [56].

The fresh and fixed samples were anatomically analyzed. The fresh samples were sectioned by hand, and the fixed samples were dehydrated in a gradual ascending ethanol series, for 2 h in each solution, embedded in Leica Historesin, and cut into 5 µm sections with a Leica RM 2245 rotary microtome. The sections were stained for histochemical tests as follows: general staining with toluidine blue (Sigma, Livonia, MI, USA), pH 6.5 and 5.8 [57]; sugars with Fehling reagent (Fluka analytical) [58] and Periodic acid Schiff (PAS) reagent (Merck, Rahway, NJ, USA) [59]; lipids with Sudan III, IV, and black reagents (Sigma) [60]; starch grains with Lugol reagent (Sigma) [61]; terpenes with NADI reagent (Sigma) [62]; tannin with vanillin-hydrochloric acid (Sigma) [63]; and protein bodies with Xylidine ponceau (Sigma) [64]. The slides were observed under a Leica DM 4500B light microscope and recorded with a Leica DFC 320 digital camera. In addition, serial longitudinal sections of the gland were obtained for a 3D reconstruction of the structure stained with toluidine blue, in After Effects (Adobe Systems, San Jose, CA, USA) [65].

The leaf glands were diaphanized by using fixed samples that were treated with 1 M sodium hydroxide for 24 h, washed in water three times, and submerged in 10% sodium hypochlorite at 50 °C until transparent. The transparent samples were dehydrated in gradual ascending ethanol series and stained with a 1% alcoholic safranin solution (Sigma). The samples were observed under a Leica MDG 34 stereomicroscope and recorded with a Leica DFC 425 digital camera.

The surface of the leaf glands was examined using scanning electron microscopy. Small leaf samples were dehydrated in a gradual ascending ethanol series for 2 h in each solution, CO_2_ critical-point dried, mounted on stubs, and gold-coated. The samples were observed and registered under a Zeiss EVO-50 (Cambridge, UK) scanning electron microscope at FMRP-USP.

The ultrastructure of leaf glands was examined using transmission electron microscopy. Small leaf samples were fixed in 4% paraformaldehyde and 2% glutaraldehyde in 0.1 M phosphate buffer, pH 7.4, for 24 h at room temperature, post-fixed in OsO_4_ in the same buffer for 2 h, dehydrated in acetone solutions, and embedded in Araldite resin (EMS 6005). Ultrathin sections of 70 nm were stained with 2% uranyl acetate and 0.3% lead citrate, at room temperature. The samples were observed and registered under a JEOL 100CX II (Akishima, Tokyo) transmission electron microscope at 80 kV, with digital imaging.

In addition, the following field observations of the plants were carried out in their collection environments: weather conditions during the time of observation such as humidity, solar intensity, and wind; the presence of visiting animals, their arrival time and departure, places visited, behavior, and interactions; and plant conditions such as signs of herbivory or discoloration. All observations were recorded between 7:00 a.m. and 6:00 p.m., for a total of 20 h of observation for each species.

## 5. Conclusions

The diversity of leaf gland types, their locations, and functions is remarkable in Rhamnaceae species, with comprehensive information available to date. The external and protuberant glands can be defined as extrafloral structured nectaries (*Colubrina glandulosa*, *Gouania polygama*, present study), food bodies (*Hovenia dulcis* [13]), or glandular trichomes (*Ceanothus* species [31]). These trichomes must still be submitted to histochemical and ultrastructural analyses to check if they secrete nectar, and thus, characterized as nectariferous. The internal glands can be defined as oil-resin secreting ducts and cavities (*Rhamnidium elaeocarpum*, present study) and lysogenic mucilage reservoirs (many species [46,47,50]). This diversity is also seen in the flowers of the species of the family [29,30], indicating that this group is a promising model for studies of the morphology and evolution of secretory structures. Among these, the atypical structure and functioning of the EFNs of *G. polygama* stands out, certainly deserving detailed studies of ontogeny, dynamics of nectar secretion, and ecological interaction.

The intricate morphology of these glands and their potential roles in plant–arthropod interactions are being described for the first time in *Colubrina*, *Gouania*, and *Rhamnidium*.

## Figures and Tables

**Table 1:** Results of the histochemical tests of the leaf glands of *Colubrina glandulosa*, *Gouania polygama*, and *Rhamnidium elaeocarpum*. Abbreviations: cyt = cytoplasm, cw = cell wall, epid = epidermis, parench = parenchyma. Symbols: (-) negative response, (+) positive response.

Stain	Target Compound	*Colubrina glandulosa*				*Gouania polygama*				*Rhamnidium elaeocarpum*		
		Glandular Epid		Glandular Parench		Glandular Epid		Glandular Parench		Epithelial Cell		Lumen
		cyt	cw	cyt	cw	cyt	cw	cyt	cw	cyt	cw	
Toluidine blue	Phenolic compounds	-	-	+	-	-	-	+	-	-	-	+
Fehling reagent	Reducing sugar	+	-	+	-	+	-	+	-	-	-	-
Lugol reagent	Starch grains	-	-	-	-	-	-	-	-	-	-	-
NADI reagent	Terpenes	+	-	-	-	+	-	+	-	-	-	-
PAS reagent	Neutral polysaccharides	+	+	+	+	+	+	+	+	+	+	-
Sudan IV	Lipids	-	+	-	-	-	-	-	-	+	-	+
Xilidine Ponceau	Protein bodies	+	-	-	-	+	-	-	-	+	-	+
Vanillin-hydrochloric acid	Tannin	-	-	-	-	-	-	-	-	-	-	-

## Data Availability

Data sharing is not applicable to this article.
